# Hospital Admissions in Children with Down Syndrome: Experience of a Population-Based Cohort Followed from Birth

**DOI:** 10.1371/journal.pone.0070401

**Published:** 2013-08-13

**Authors:** Patrick Fitzgerald, Helen Leonard, Terri J. Pikora, Jenny Bourke, Geoffrey Hammond

**Affiliations:** Telethon Institute for Child Health Research, Centre for Child Health Research, University of Western Australia, Crawley, Western Australia, Australia; IGBMC/ICS, France

## Abstract

**Objective:**

Children with Down syndrome, the most common genetic cause of intellectual disability, are prone to multiple and varied health-related problems. This study describes patterns of hospitalisations for children and young people with Down syndrome in Western Australia.

**Methods:**

Birth records were linked to the Western Australian population-based Intellectual Disability database to identify all children born with Down syndrome in Western Australia between 1 January, 1983 and 31 December, 1999. These records were linked to the Hospital Morbidity Data System to provide information on all hospitalisations up to 31 December, 2004. Hospitalisation data, coded using ICD-9CM or ICD-10 (v0.5) were grouped into clinically relevant categories using the primary diagnosis. Rates of hospital admission for all and specific diagnoses were expressed in 1000-person-years at-risk and median age at first admission and length of stay were calculated.

**Results:**

Of the 405 children, 395 had one or more hospital admissions, totalling 3786 admissions for all children and an estimated 39.5 person-years in hospital. On average, children were admitted 9.7 times, with an estimated rate of 757.2 admissions per 1000pyr (95% CI: 680, 843). A quarter of all admissions occurred in the first year of life. The average hospital length of stay was 3.8 days (95% CI: 3.7, 4.1). Upper respiratory tract conditions affected the most children (58.5%) and accounted for 12.1% of all admissions. Other disorders which affected a high percentage of children were ear/hearing conditions (50.6%), disorders of the oral cavity (38.0%) and lower respiratory tract conditions (37.5%). Overall, children with Down syndrome were hospitalised at a rate five times (95% CI = 4.3–6.2) that of the general population.

**Conclusion:**

Children with Down syndrome are at increased risk of morbidity for varied causes underlining the importance of comprehensive and targeted primary care for this group.

## Introduction

Down syndrome is the most common genetic cause of intellectual disability and, despite increasing prenatal diagnosis and subsequent termination of pregnancy [1], [2], for the past twenty years in Western Australia the prevalence has remained relatively stable at approximately 1/1000 live births [3]. Children with Down syndrome are prone to multiple health-related problems [4], including congenital heart defects [5], [6], [7], [8], congenital gastrointestinal problems [5], [6], [8], ear and hearing impairments [9], thyroid disease, childhood leukaemia [10], [11], [12], [13], and immune-related disorders (including coeliac disease, thyroid and diabetes mellitus) [14]. In addition they have been found to have an increased risk of respiratory infections and resulting hospitalisations, most commonly for pneumonia and bronchiolitis [15], [16]. Furthermore, the presence of congenital heart disease has been shown to increase both the risk of hospitalisation overall [6], [15] and for respiratory infections [16]. Changes in clinical treatments, including access to cardiac surgery, have been shown to improve both survival rates [7], [17], [18] and overall health [17]. The high incidence of infections, haematological malignancies and immunity-related disorders during the early years of children with Down syndrome may be a consequence of congenital barriers to development of T and B lymphocytes [19], [20].

The current study describes patterns of hospitalisations for children and young people with Down syndrome in Western Australia, including rates of hospitalisation, reason for admissions and length of stay.

## Methods

### Data

Birth records from the Midwives Notification System [21] of Western Australia were linked to the Western Australian population-based Intellectual Disability (IDEA) database [22] to identify all children born with Down syndrome in Western Australia (WA) between 1 January, 1983 and 31 December, 1999. Resulting records were then linked to the Hospital Morbidity Data System (HMDS) of the Health Department of Western Australia [23], which records diagnostic, procedural and discharge information on all hospital admissions in WA and has previously been used to measure morbidity in children with intellectual disability [24]. Using HMDS data, children were followed from birth to December 31, 2004, or until death.

Hospital morbidity data use Australian standard ICD-9CM or ICD-10 (v0.5) diagnostic codes (changes in coding conventions occurred in July 1999 in WA). Each record consisted of a single primary and up to 19 secondary diagnostic codes and was grouped into clinically relevant categories using the primary diagnostic category or a specific subdiagnosis where appropriate or required [24], [25]. The general categories used were: respiratory (further divided into upper, lower, and general), ear and hearing, oral cavity/teeth, cardiac conditions, eye and vision systems, musculoskeletal, renal/genitourinary systems, digestive systems, skin/augmentary systems, endocrine/metabolic/immune, central nervous system, blood disorders, external causes, and leukaemia. Admissions with a primary (ie., first) diagnosis of “Down syndrome” (ICD-9CM code 758.0, n = 58), were recoded to reflect the secondary or most likely cause of hospitalisation. Secondary diagnoses were otherwise only used to ensure complete ascertainment of the prevalence of congenital cardiac defects in the population.

In addition to recorded diagnosis of selected diseases, procedures or interventions, coded using ICD-9CM [26] standards, were also available for each admission. We were particularly interested in assessing the prevalence of invasive cardiac procedures in this population. Open heart surgery was not available in WA before 2000; prior to this, simple heart surgery was performed in WA and complex cases were referred to a specialist unit at the Melbourne Children's Hospital (3,400 km distant) if feasible. As information on cardiac procedures conducted in Melbourne was not available in the HMDS data, a cardiac procedure was inferred if, before 2000, a child had been admitted with a cardiac diagnosis and co-occurring aftercare codes.

In order to assess the degree of additional morbidity experienced by children and adolescents with Down syndrome relative to the WA child and adolescent population, we compared rates observed in our cohort to those described in a report on common specific conditions using HMDS population-based data from 1995 [25]. The hospitalisation rates from this report were used as comparators to the Down syndrome cohort in the current study using a rate ratio estimate (similar diagnostic categorizations were used in both studies). The approximate time at risk for the WA population was provided in the documentation.

### Statistical methods

Rates of hospital admission were expressed in 1000-person-years at-risk (1000 pyr) and estimated by dividing the number of admissions by the corresponding total person-years-at-risk (calculated as the time out of hospital and before death). Unless otherwise specified, age is reported as median age at first admission. Poisson regression analysis was used to assess rate change throughout the life course correcting for calendar time and child gender [27]. Cox regression was used to compare time to first hospitalisation by gender. Mortality rates by gender were compared using the log-rank test [28]. Stata™ (v11) [29] was used for all inferential analyses while Microsoft Excel® [30] was used for coding of diagnostic categories.

Ethical approval for the study was provided by the University of Western Australia and the Human Research Ethics Committee for the Department of Health, WA. Informed consent was not required by participants as the data were provided as part of a de-identified population dataset through the Western Australian Data Linkage System and consent was considered impractical by the Ethics Committee.

## Results

### Cohort characteristics

Of the 423,033 children born in WA between 1983 and 1999, 405 were born with Down syndrome. Of the 251,298 male children (51.4% of the total) born during this period, 233 of the Down syndrome children (57.5%) were males. The average live birth rate was 0.97 per 1000 live births (95% CI: 0.88, 1.08) and did not vary by gender. Individual follow-up periods ranged from a minimum of 0.21 years to a maximum 21.86 years (median 12.59 years). During the period of observation, 36 of the 405 children (8.9%) died between 1983 and 2004.

### Overall rates of hospital admission and length of stay

Of the 405 children, 395 had one or more HMDS admissions. In total, the HMDS data comprised 3786 admissions, spanning 4999.9 person-years of observation. On average, children were admitted 9.7 times over their period observed, with an estimated rate of 757.2 admissions per 1000 pyr (95% CI: 680, 843). These 395 children accumulated an estimated 39.5 person-years in hospital.

**Figure 1:**
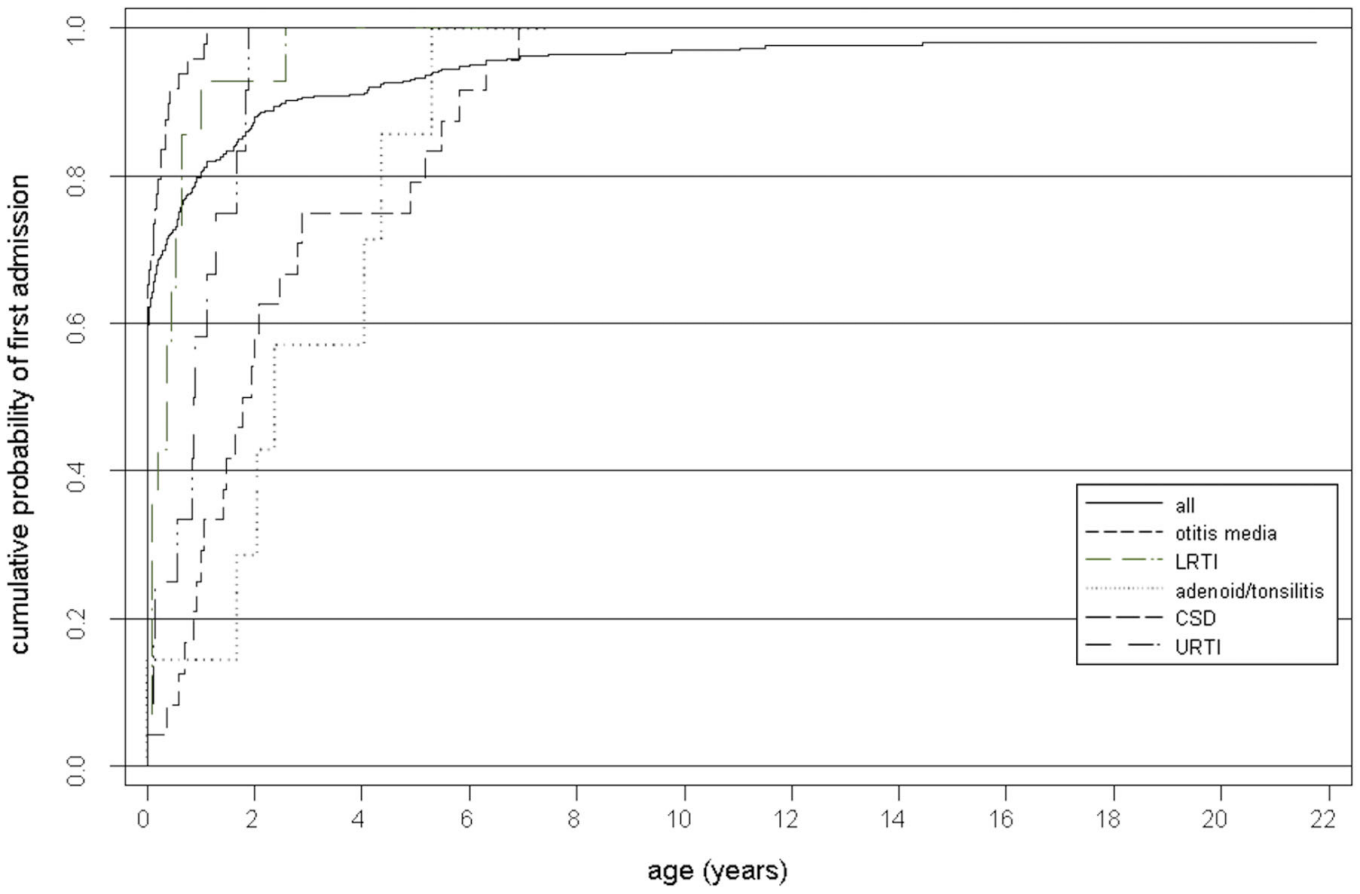
Cumulative probability of first admission by age and most common diagnoses.

Two hundred and fifty five (63%) children were admitted in their first month and 323 (79%) in their first year of life (Figure 1). Nearly a quarter of admissions (937, 24.7% of the total number) occurred in the first year of life with approximately one third of those (344, 9.1%) occurring in the first month. Overall the rate of hospital admission declined considerably with age (1.1% decline per month, IRR: 0.99, 95%CI: 0.986–0.992, *p*<0.001) after adjusting for gender and year of admission. Time to first admission was similar for both genders (HR: 0.92, 95% CI: 0.75–1.12, *p* = 0.39).

The average hospital length of stay (LoS) was 3.8 days (95% CI: 3.7, 4.1), with a maximum of 112, but durations of one day or less were recorded in 56.4% of admissions and 95.3% of admissions were of 14 days or less. Comparing 1983 to 1999, there was a general decline in LoS, both in median days (7 to 1) and mean days (11 to 2, F: (258, 1): 37.1, *p*<0.001).

### Diagnostic Groups

Over the follow-up period, a substantial proportion of children were hospitalised for numerous diagnoses, with each child admitted for an average of 2.93 conditions (median: 4, maximum 20). In addition, multiple comorbidities were recorded on admission with up to 10 different groups simultaneously.

Admission frequencies and rates by diagnostic group (and specific subdiagnoses) for all hospital admissions are presented in Table 1. More children (58.5%) had been admitted for an upper respiratory tract condition than for any other major diagnostic group and accounted for 12.1% of all admissions. Other disorders, which affected a high percentage of children, were ear/hearing conditions (50.6%), disorders of the oral cavity (38.0%) and lower respiratory tract conditions (37.5%). Ear/hearing conditions occurred in a large percentage of individuals and also accounted for a high proportion (14.5%) of all admissions. However, while admissions for leukaemia constituted a relatively large proportion of the total number of admissions (7.4%), they affected a relatively small proportion of children (4.0%). Disorders with a relatively low median age at first admission included endocrine/metabolic conditions (largely attributable to neonatal jaundice), cardiac (largely driven by cardiac septum defects), and lower (driven by acute bronchiolitis) and general respiratory tract disorders (median ages: 4 days, 4 months, 10 months, and 1.1 years, respectively). Primary diagnoses with a later median age at first admission included disorders of the musculo-skeletal system (median age: 5.3 years) and oral cavity/teeth (median age: 7.8 years).

**Table 1:** Primary diagnosis by group, ordered by median age at first admission.

Diagnostic Group	*Subdiagnoses*	Children (%)	Admissions (%)	Rate/1000 PYAR^1^ (95%CI)	Median age first admission
**Upper Respiratory Tract**		**237 (58.5)**	**457 (12.1)**	**91.4 (79.7, 104.8)**	**2.8 Years**
	*Tonsils/Adenoids*	139 (34.3)	158 (4.2)	31.6 (27.0,36.8)	5.1 Years
	*Croup*	67 (16.5)	116 (3.1)	23.2 (19.3, 27.7)	1.7 Years
	*Sleep Apnoea*	21 (5.2)	26 (0.7)	5.2 (3.5, 7.5)	8.6 Years
**Lower Respiratory Tract**		**152 (37.5)**	**405 (10.7)**	**81.0 (65.0, 100.9)**	**1.1 Years**
	*Pneumonia*	100 (24.7)	198 (5.2)	20.0 (16.3, 24.2)	2.4 Years
	*Acute Bronchiolitis*	57 (14.1)	89 (2.4)	17.8 (14.4, 21.8)	5 Months
	*Asthma*	28 (6.9)	64 (1.7)	12.8 (9.9, 16.2)	2 Years
**Respiratory Tract General**		**82 (20.2)**	**146 (3.9)**	**29.2 (21.5, 39.7)**	**10 Months**
	*Congenital Anomalies*	6 (1.5)	10 (2.5)	2.0 (1.0, 3.6)	1 Year
**Ear and Hearing**		**205 (50.6)**	**550 (14.5)**	**110.0 (96.0, 126.1)**	**2.5 Years**
	*Otitis Media*	194 (47.9)	457 (12.1)	91.4 (83.3, 100.1)	2.5 Years
**Oral cavity/Teeth**		**154 (38.0)**	**215 (5.7)**	**43.2 (37.4, 49.9)**	**7.8 Years**
	*Congenital Oral Cavity Conditions*	28 (17.6)	33 (0.9)	6.6 (4.6, 9.2)	6.7 Years
	*Caries*	101 (24.9)	123 (3.2)	24.6 (20.5, 29.3)	6.7 Years
**Cardiac Conditions**		**108 (26.7)**	**243 (6.4)**	**48.6 (42.8, 55.0)**	**4 Months**
	*Cardiac Septum Defects (CSD)*	92 (22.7)	189 (5.0)	37.8 (32.7, 43.4)	3 Months
	*Patent Ductus Arteriosus (PDA)*	14 (3.5)	15(4.0)	3.0 (1.7, 4.8)	1.2 Years
	*Heart Failure*	12 (3.0)	18 (4.8)	3.6 (2.2, 5.6)	7 Months
	*Pulmonary Hypertension*	7 (0.2)	4 (1.0)	0.8 (0.3, 1.9)	1.1 Years
**Infections**		**321 (79.3)**	**1243 (32.8)**	**24.9 (23.5, 26.3)**	**1.2 Years**
	*Respiratory Infections*	213 (52.6)	570 (15.1)	11.4 (10.5, 12.4)	1 Year
	*Otitis Media*	194 (47.9)	457 (12.1)	91.4 (83.3, 100.1)	2.5 Years
	*Enteric or Diarrhoea-causing Infections*	63 (15.6)	82 (2.2)	16.4 (13.1, 20.3)	2 Years
**Digestive System**		**153 (37.8)**	**380 (10.0)**	**76.0 (68.6, 83.9)**	**1.5 Years**
	*Congenital digestive system abnormalities*	28 (6.9)	62 (1.6)	1.2 (9.6, 15.8)	4 Days
	*Upper Digestive Tract*	22 (5.4)	31 (0.8)	6.2 (4.3, 8.7)	5.3 Years
	*Lower Digestive Tract*	37 (9.1)	110 (2.9)	22.0 (18.2, 26.4)	4.1 Years
	*Enteric or Diarrhoea-causing Infections*	63 (15.6)	82 (2.2)	16.4 (13.1, 20.3)	2 Years
**Eye/vision Systems**		**86 (21.2)**	**161 (4.3)**	**32.2 (27.5, 37.4)**	**2.7 Years**
	*Disorders to eye movement & support*	74 (18.3)	118 (3.1)	23.6 (19.6, 28.3)	3.1 Years
	*Congenital eye anomalies*	19 (0.5)	16 (4.0)	3.8 (2.4, 5.8)	2.9 Years
**Renal/genitourinary System**		**64 (15.8)**	**129 (3.4)**	**24.8 (16.5, 37.3)**	**4.7 Years**
	*Congenital Anomalies*	23 (5.7)	42 (1.1)	8.4 (6.1, 11.2)	4.5 Years
	*Bladder*	13 (3.2)	39 (1.0)	7.8 (5.6, 10.6)	3.1 Years
	*Male Reproductive*	31 (7.7)	37 (1.0)	7.4 (5.3, 10.1)	6.4 Years
**External Causes**		**65 (16.0)**	**76 (2.0)**	**15.2 (12.1, 18.9)**	**3.8 Years**
	*Poisoning*	11 (2.7)	11 (2.7)	2.2 (1.2, 3.8)	4.5 Years
	*Foreign Bodies Needing Removal*	11 (2.7)	12 (0.3)	2.4 (1.3, 4.1)	4.2 Years
	*Burns*	6 (1.5)	6 (0.2)	1.2 (0.5, 2.5)	3.1 Years
**Musculoskeletal**		**67 (16.5)**	**103 (2.7)**	**20.6 (16.9, 24.8)**	**5.3 Years**
	*Congenital musculoskeletal conditions*	10 (2.5)	11 (0.3)	2.2 (1.2, 3.8)	4.8 Years
	*Fractures of an extremity*	9 (2.2)	12 (0.3)	2.4 (1.3, 4.1)	7.8 Years
	*Bone and rheumatic disorders*	5 (1.2)	11 (0.3)	2.2 (1.2, 3.8)	6.6 Years
	*Wounds, Abrasions, or lacerations*	16 (4.0)	19 (0.5)	3.8 (2.4, 5.8)	5.2 Years
**Skin/augmentary System**		**47 (11.6)**	**63 (1.7)**	**12.8 (9.2, 17.9)**	**4.8 Years**
	*Cyanosis*	5 (1.2)	6 (0.2)	1.2 (0.5, 2.5)	9 Months
	*Cellulitis*	10 (2.5)	12 (0.3)	2.4 (1.3, 4.1)	3.9 Years
**Endocrine/metabolic/immune**		**34 (8.4)**	**60 (1.6)**	**14.0 (7.6, 25.8)**	**4 Days**
	*Fetal Neonatal Jaundice*	17 (4.2)	18 (0.5)	3.6 (2.2, 5.6)	1 Day
	*Immunoglobulin Deficiencies*	2 (0.5)	25 (0.7)	5.0 (3.3, 7.3)	4.8 Years
**CNS Disorders**		**20 (4.9)**	**32 (0.8)**	**6.4 (4.5, 8.9)**	**1.5 Years**
	*Epilepsy*	5 (1.2)	8 (0.2)	1.6 (0.7, 3.0)	1.6 Years
	*Non-febrile Convulsions*	5 (1.2)	7 (0.2)	1.4 (0.6, 2.8)	1.1 Years
**Blood Disorders**		**16 (4.0)**	**33 (0.9)**	**6.6 (4.6, 9.2)**	**3.1 Years**
	*Anemia*	7 (1.7)	9 (0.2)	1.8 (0.9, 3.3)	2.1 Years
	Agranulocytosis	4 (1.0)	10 (0.3)	2.0 (1.0, 3.6)	4.7 Years
**Leukaemia**		**16 (4.0)**	**281 (7.4)**	**56.2 (28.5, 110.9)**	**4.1 Years**
**Generalised Infections**		**63 (15.6)**	**88 (2.3)**	**17.6 (14.2, 21.5)**	**2.4 Years**
**Other Non-clinical**		**30 (7.4)**	**36 (1.0)**	**7.2 (5.0, 10.4)**	**0.6 Years**
**Other Diagnoses**		**175 (43.2)**	**245 (6.5)**	**49.0 (42.7, 56.2)**	**1 day**
**Total** 2		**405**	**3,786**	**757.2 (680.2, 843.0)**	**2 Years**

1 PYAR: person-years-at-risk of admission;

2 includes 10 children with no record of admission on the HMDS.

The five most common individual primary admission diagnoses as based on largest number of admissions corresponded to the following ICD-9CM codes: 381, 382 (otitis media); 466 (lower respiratory tract infection); 461.2, 462, 463, 464, 464.2, 464.21, 464.3, 464.4, 465.9 (upper respiratory tract infection); 745, 746 (cardiac septum defects); and 474, 474.1, 474.11, 474.12, 478, 478.1(adenoid/tonsillitis). Otitis media, lower respiratory tract infection, upper respiratory tract infection, cardiac septum defects, and adenoid/tonsillitis constituted 12.1%, 10.7%, 5.0%, and 4.2% of admissions, respectively. Figure 1 shows that the majority of these disease codes occurred relatively early in the life course, with more than 80% of admissions attributable to these causes occurring before the age of two years. By the age of 8 years, more than 90% of the admissions had occurred.

**Figure 2:**
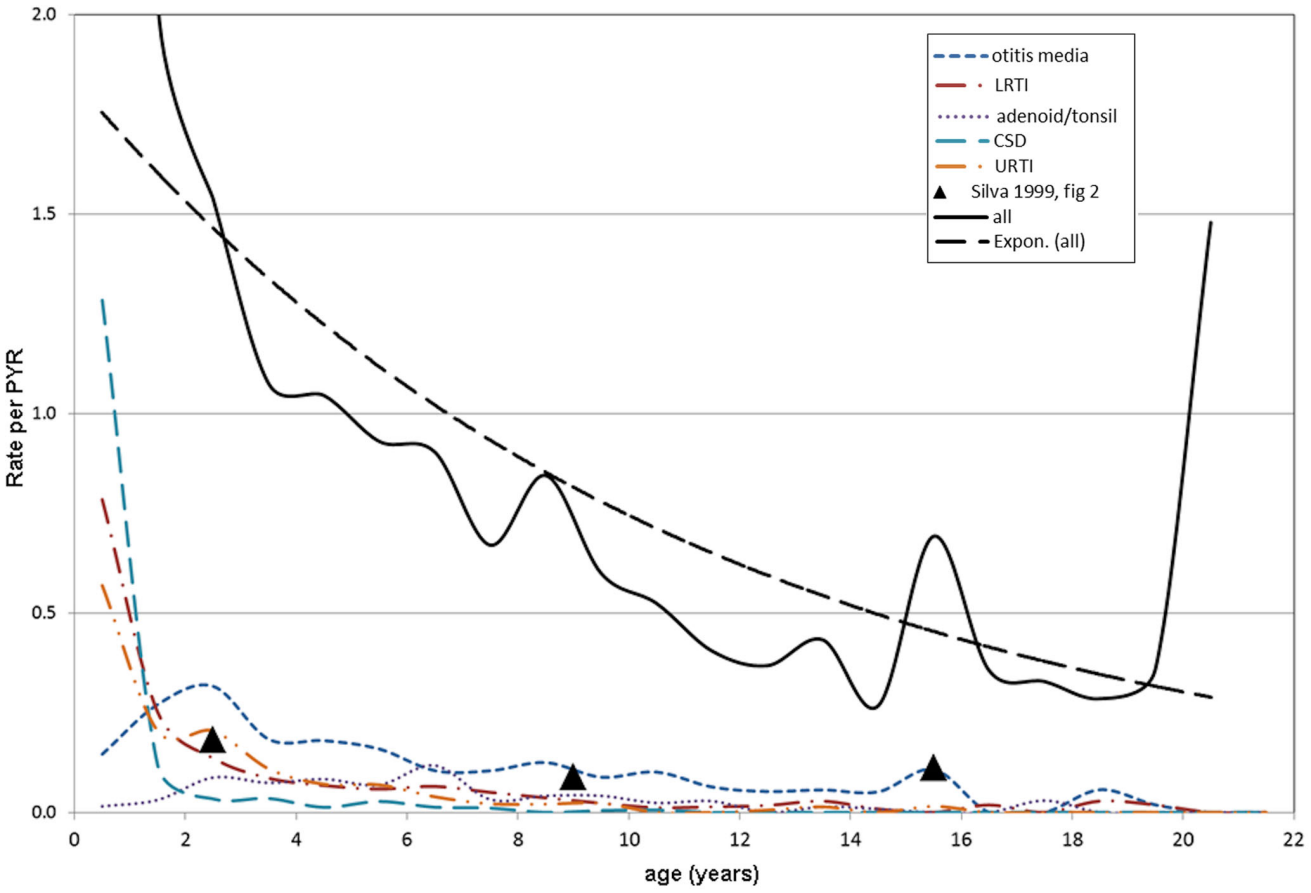
Rates of hospital admission by year of age - most common diagnoses, all admissions, predicted rate.

Figure 2 displays the overall hospitalisation rates by patient age as well as rates for the five common diagnoses described above. Although the overall rate of admissions decreased with age, three transitory increases in overall rates were observed: one at the age of nine, another at the age of 15, and a third at the age of 20 years. Increases at nine and 15 corresponded to observed increases in the rate of otitis media. The increase observed at 20 years of age was attributable to a combination of low total time of observation and a single patient admitted repeatedly for cancer treatments during that period.

The following subsections provide more detail on admission diagnostic groups and subdiagnoses.

#### Respiratory tract conditions

Respiratory tract infections (upper, lower, and general) accounted for a quarter of all admissions (26.7%) and were also associated with a high rate of infection (201.6/1000 pyr). Upper respiratory tract conditions affected more than one half of all children (58.5%), followed by lower (37.5%) and general (20.2%) (Table 1). The median age at admission was 2.8 years for upper respiratory tract, 1.1 years for lower respiratory tract, and 10 months for general respiratory tract admissions (reported in Table 1). Admission rates for respiratory conditions were similar for males and females.

Prevalent subdiagnoses for upper respiratory tract infections included infections of the tonsil and/or adenoid glands which resulted in admissions for 139 children (34.3%) at a rate of 31.6 per 1000 pyr with a median age of 5.1 years at first admission. Croup was also identified as a prevalent diagnostic category (affecting 16.5% of children, rate: 23.2 per 1000 pyr, median age at first admission: 1.7 years). Sleep apnoea (affecting 5.2% of children, rate: 5.2 per 1000 pyr, median age at first admission: 8.6 years) also accounted for a small proportion of upper respiratory admissions.

Common lower respiratory tract subdiagnoses included pneumonia (24.7% of children affected at a rate of 20.0 per 1000 pyr with a median age of 2.4 years at first admission) and acute bronchiolitis (affecting 14.1% of children, rate: 17.8 per 1000 pyr, median age at first admission: five months). Asthma was a less prevalent lower respiratory tract diagnosis: it was recorded as the primary admission for 6.9% of individuals studied (rate:12.8 per 1000 pyr, median age at first admission: 2.0 years). Few common diagnostic categories were observed for general respiratory tract conditions with congenital anomalies (1.5%, rate: 2.0 per 1000 pyr, median age at first admission:1 year) being the largest single cause of morbidity.

#### Ear and hearing conditions

Ear and hearing conditions were common among the cohort with one half (50.6%) of individuals having an admission and 14.5% of all admissions related to this condition. Rates were similar for males and females. Median age at first admission for these conditions was 2.5 years. Otitis media was a highly prevalent subdiagnosis, accounting for 457 admissions and occurred at least once in 47.9% of the studied population. The median age at first admission for otitis media was 2.5 years. For the majority of those with an otitis media diagnosis (86.1%), myringotomy and tube insertion was performed.

#### Oral cavity and teeth conditions

One hundred and fifty four children (38.0%, rate of 43.2 per 1000 pyr) were admitted for disorders of teeth and the oral cavity with a median age at first admission of 7.8 years. Rates were similar for males and females. Of the 154 individuals, 28 (17.6%) had oral cavity disorders which could be related to congenital conditions, including cleft-palate, missing teeth, crowding of teeth and malocclusion. One hundred and one individuals (24.9% of the total sample) were admitted for caries (rate: 24.6 per 1000 pyr, age at first admission: 6.7 years).

#### Cardiac conditions

Whilst only 26.7% of the cohort had a primary cardiac diagnosis (Table 1), taking into account either CSD or PDA as a primary or secondary diagnosis, 165 individuals (40.7%) had a diagnosis for a cardiac defect (Table 2). Adding in other potential codes which might be recorded in conjunction with cardiac complications, the total number of individuals with a cardiac diagnosis was just under half (190 individuals, 46.9%) There was significant overlap between cardiac defect diagnoses and other cardiac conditions. Records of surgical procedures undertaken and/or post-operative admission codes were identified for 25.9% of children (105 individuals).

**Table 2:** Proportion of children with congenital cardiac defects and resulting recorded surgical procedures.

Condition	Prevalence^1^		Surgical Procedure	
	Recorded as primary or secondary admission (%)	Recorded as primary admission (%)		Recorded as procedure code (%)
**congenital defect**			**repair**	
*CSD* 2	154 (38.0)	92 (22.7)	*heart*	51 (12.6)
*PDA* 2	56 (13.8)	14 (3.5)	*cardiac vessel*	25 (6.2)
*Either CSD or PDA*	165 (40.7)	103 (23.4)	*Either heart or cardiac vessel*	72 (17.8)
**other**			**other**	
*heart failure*	27 (6.7)	12 (3.0)	*CABG* 3	5 (1.2)
*pulmonary hypertension*	68 (16.8)	4 (1.0)	*CC* 4	54 (13.3)
*other*	48 (11.9)	9 (2.2)	*other*	5 (1.2)
*Any of the three aforementioned conditions*	139 (34.3)	23 (5.7)		
*Any cardiac condition*	190 (46.9)		*Any cardiac repair*	105 (25.9)

1 n = 405;

2 subgroups are not mutually exclusive, eg.: 20 children were diagnosed with both CSD and PDA, 4 were admitted;

3 CABG: coronary artery bypass graft;

4 CC: cardiac catheterization.

#### Digestive system

A total of 153 children (37.8%) were admitted with digestive system disorders on 380 occasions. Twenty eight individuals (6.9%) were admitted for congenital anomalies (at a median age of 4 days) leading to an additional 62 related hospitalisations. Another large subgroup of GI admissions was attributable to enteric or diarrhoea causing infections (63 children, 82 admissions, rate: 16.4 per 1000 pyr, median age at first admission: 2.0 years). Rates of GI infections were similar for males and females. However, lower digestive tract conditions accounted for almost three times more admissions than upper digestive tract conditions.

#### Infections

Almost one third (32.8%) of the 3786 admissions were for infections, affecting 321 (79.3%) children with an admission rate of 24.9 per 1000 pyr. The median age at first admission was 1.2 years and rates for males and females were similar. Common types included respiratory infections (affecting 213 children (52.6%) who were admitted on 570 occasions), and enteric or diarrhoea-causing infections (affecting 63 children (15.6%) who were admitted on 82 occasions).

#### Less common admission diagnoses

The remaining diagnostic groups and specific subdiagnoses listed in Table 1 represent less commonly recorded conditions. However, 16 children (4.0%) were diagnosed with leukaemia, the only cancer type diagnosed in this cohort. Ten of these children were diagnosed before the age of five years and three were diagnosed over the age of 18 years. These 16 children were admitted on 281 occasions, including 215 for chemotherapy. Four children died from leukaemia or related complications, all under the age of 10 years.

### Length of stay

**Figure 3:**
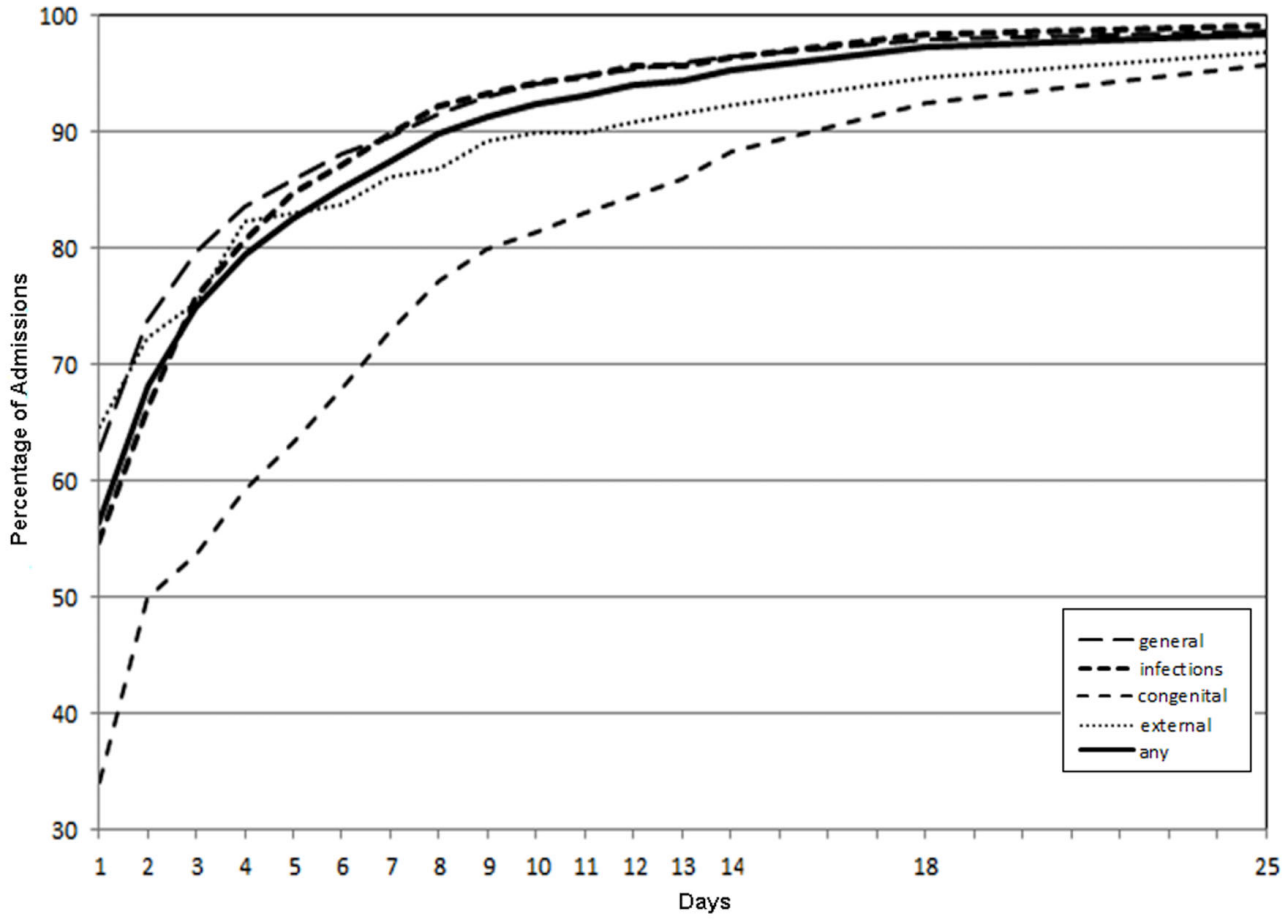
Cumulative probability of hospital Length of Stay, by admission type.

The length of hospital stay by primary diagnostic group is shown in Figure 3. Although most hospitalisations were quite short, with 70% lasting 2 days or less, there was heterogeneity across types of admission. One notable difference was in admissions for congenital anomalies, which were longer, with about 30% of admissions lasting over a week (a mean of 7.3 days and median of 3 days).

### Comparison with general population

Comparisons between the Down syndrome cohort and published rates pertaining to the WA child population are presented in Table 3. Overall, the rate of admission was significantly increased for all of the hospitalisation groups listed.

**Table 3:** Rates of common diagnoses in the Down Syndrome Cohort and as reported in the WA population by Silva et al. (1999).

Diagnosis Group	Down Syndrome Cohort Rates (per 1,000 pyr)	Silva (1999) Population based Rates (per 1,000 pyr)	Rate Ratio*
Infections	248.6	41.0	6.1 (4.4–8.7)
Respiratory System	197.4	11.2	17.9 (9.8–36.5)
Digestive System	71.4	9.5	7.1 (3.6–15.4)
Dental/Oral	43.0	8.1	5.4 (2.5–13.2)
Neoplasms	55.8	4.1	14.0 (5.2–53.2)
Renal/genitourinary	25.8	5.0	5.2 (2.0–17.3)
Injury and Poisoning	25.6	18.6	1.4 (0.7–2.6)
Overall Hospitalisation Rate	757	147	5.2 (4.3–6.2)

Significant differences in the specific diagnoses constituting each group described above were also observed. For example, for the Down syndrome cohort, 52% of infectious disease admissions were attributable to otitis media as opposed to 24% in the general population and 30% to chronic tonsillar conditions as opposed to 21% in the general population. For dental disorders in the Down syndrome group, 57% of admissions were attributable to dental caries as opposed to only 32% in the general population. Leukaemia accounted for 100% of the cancer types experienced in the Down syndrome population as opposed to 25% in the general childhood population [31]. Asthma admissions only constituted 2% of the respiratory morbidity in the Down syndrome population as opposed to 48% in the general population.

### Mortality

Thirty-six children died during the study period. Death rates fell dramatically with age, and rates were similar for males and females (hazard-ratio (HR: 1.75 (95% CI: 0.90, 1.11), log-rank test: *p* = 0.156). There was strong evidence that death rates reduced over calendar year of birth (HR (per year): 0.845 (95% CI: 0.776, 0.920), log-rank test *p* = 0.004).

There was an increased risk of mortality for children with a diagnosed cardiovascular condition compared with those without (HR: 4.35, 95% CI: 2.20, 8.61, *p*<0.001) and this risk had changed over time when comparing the earlier years (1984–1994) with the later years (1995–2004) (HR: 5.89, 95%CI: 1.41, 24.59, *p* = 0.015).

## Discussion

This population-based study comprehensively describes hospitalisations of children with Down syndrome born in WA between 1983 and 1999 and followed until 2005. Supported by earlier studies [4], [6], [16], [17], [32] our results clearly infer an elevated morbidity for children with Down syndrome, with hospitalisation rates five times that of the general population [25]. Rates for specific conditions such as infections and neoplasms and respiratory and digestive disorders were particularly elevated. Consistent with previous research [15], [16], we also found that children with Down syndrome were hospitalised early and often. The majority, 80% of children in our cohort, had been admitted to hospital within the first year of life, with a quarter of all hospitalisations occurring within that period and about a third of these within the first month.

On average, each child with Down syndrome was admitted nearly ten times, summing to a total of almost 40 person-years in hospital for only 405 individuals, representing significant and prolonged morbidity over time. While the overall rate of hospitalisation declined considerably with age, it did not change over the study period. While for most diagnostic groups there was little difference in hospitalisation rates by gender, cardiovascular admissions did occur more often in females, as has been reported previously [15], [18]. Our results broadly concur with those of another Australian study where it was reported that 27% of admissions occurred in the first year of life and that 72% of all admissions were among those aged less than 5 years of age [15].

Our results also highlight a large reduction in the length of stay during the period analysed. Improvements in medical care as well as increased availability to outpatient services are likely to have brought about some of the changes observed [17]. Overall the average length of stay reported in two other studies [15], [16] is similar to our population, but their numbers of admissions are likely underestimates of the true extent of admissions in a total Down syndrome population. Moreover, neither study followed individuals over the time period or the duration of our study.

As confirmed by our findings, children and young people with Down syndrome are at increased risk of infection, especially early in the life course, likely due to structural abnormalities and an immature immune system [33]. Similar to previous research [16], the greatest morbidities in our study were respiratory tract conditions. However early surgical intervention and improved treatments for cardiac defects may be leading to a decrease in respiratory infections over the long term [17].

Children with Down syndrome are predisposed to middle ear infections due to Eustachian tube abnormalities [34], [35] as well as lowered immunocompetence [36]. In our cohort, half of the children had at least one ear/hearing admission, with the vast majority of these due to otitis media, a proportion higher than that reported in the earlier Australian study [15]. Admission rates for this condition were also considerably higher among our cohort compared to the population rates reported for both pre-school and primary school aged children [31]. Our finding of a high rate of ear and hearing problems in the Down syndrome population is consistent with previous WA research using parental report [4], [17]. Myringotomy and tube insertion among our Down syndrome cohort was much more common than in the general population [31], with 86.1% of individuals with otitis media undergoing this procedure. Chronic and severe ear infections may lead to hearing loss and have been found to impact on all facets of the young person's life including cognitive and language development and socialisation [9], [37], [38]. It is recommended that children with Down syndrome undergo regular screening early in life to identify any hearing impairments, management of which will assist in reducing any secondary speech problems that may result [9], [39], [40].

Cardiac defects have been reported to be the most common and serious birth defect among children with Down syndrome [5], [6], [18], [33]. Similar to previous research [6], [7], [16], almost half of our population had been admitted for a cardiac condition, with the majority of these for CHD. Our estimates are higher than those reported by Hilton et al. [15] although their data spanned a much shorter time period and was unable to capture an individual's full hospitalisation record. In addition, their data were collected from a single hospital that did not perform any cardiac surgery, and so patients with CHD or associated complications were unlikely to be taken there for treatment.

It has been suggested that the orofacial deformities associated with Down syndrome combined with poor dental hygiene may contribute to the prevalence of dental disease in this population [33]. A further issue relating to the high hospitalisation rates may be the difficulty in cooperating with dental treatment in the chair [9], a likely reason for the two-fold increase in dental hospitalisations for those with any intellectual disability [41]. Over a third of the children in our cohort had at least one admission for dental treatment; two thirds of admissions were due to caries, contrasting with less than one third for the general WA childhood population [25], [42]. The median age of onset for dental admissions was 6.7 years for the Down syndrome population as compared to the peak admission rate in adolescence for the WA child population [31]. There is the need for dental screening and dental care programs that target young people with Down syndrome that, along with routine and regular oral hygiene practice, may assist in reducing the incidence of preventable dental disease [33], [39]. Early, frequent dental visits and scaffolding may also increase cooperation levels and ensure better dental hygiene.

As did others [10], [11], [12], [13], we found that rates of leukaemia admission among young people with Down syndrome were much higher than in the general population [31], [43]. Each child with leukaemia was admitted an average of 13 times with the majority for chemotherapy treatment. These individuals therefore represent a large proportion of the hospitalised adolescent population.

While Down syndrome births made up only 1.6% of WA children born with congenital anomalies between 1983 and 1999 [3], the common admission diagnoses concur with those in a broader study of WA children with birth defects [44]. Within the group of children with major birth defects, those with Down syndrome experienced the highest number of admissions per child despite representing only 2.9% of all admissions for children with birth defects [44].

Our research demonstrates that, although the rates for most diagnoses were disproportionally higher among the Down syndrome cohort, rates for injury and poisoning were similar to the general WA child population. These results are especially informative to policy makers and health service administrators as they provide detailed insight into the population health burden associated with Down syndrome. Our results also augment estimates from a previously published economic evaluation, which were derived using patient reports of hospitalisations in Western Australia [45]. Specifically, our finding that the majority of children have been admitted by the age of five years concur with the findings that most of the medical costs associated with Down syndrome relate to clinical management early in life [45]. Benefits of early surgical treatment include increased lifespan as well as improved quality of life, likely as a function of increased independence and the ability to engage in more social activities.

While a number of previous studies have described the health conditions occurring in Down syndrome, few have had access to comprehensive, longitudinal population-based hospitalisation records for children with Down syndrome. Rather, previous studies have relied on small clinical samples, case studies (e.g. [8], [15], [46]), disease registries (e.g. [47]), or parent-report (e.g [17], [32]). A further strength of our study is linkage between administrative hospital datasets and a population-representative database on intellectual disability [48], allowing identification of all children with Down syndrome rather than relying on ascertainment from coded hospital records (eg. [10], [11], [16], [49]). Due to the statutory requirements for recording all hospitalisations in WA, the availability of complete hospitalisation records for the entire cohort allowed a detailed description of the patterns of morbidity experienced by children and young people with Down syndrome. In addition, our study provides a comprehensive picture in a single population of overall hospitalisation rates (including rates for a range of diagnostic groups), length of stay, and mortality. This contrasts with previous studies using population-based data which have had a narrow focus on either specific disease types (e.g. [10], [12], [13], [49], [50]) or on mortality outcomes (e.g. [11], [18]).

Some limitations of our study warrant mention. We had no access to individual-level data on the non-Down Syndrome population, hampering cross-comparisons, although we did compare our results with previously published population based data spanning a year within our period of observation. [25], [31] Length of follow-up varied from a minimum of 5 years to a maximum of 21 years, indicating that prevalence estimates for disorders typically occurring early in the life course are likely to be more accurate and reliable than for disorders typically occurring later in the life course. This may have prevented our ability to report fully on some conditions more likely to manifest in early adulthood, such as mental health disorders. In addition, we were unable to account for changes over time in medical and treatment care as well as admission practices. As, prior to 2000, children had to be transferred to another Australian state for the performance of all major cardiac surgery, we have made assumptions based on the presence of certain diagnosis and treatment codes in the current dataset. Future work in this area could focus on surgical care among this population using retrospective record review to determine specific diagnoses and treatments. Unlike other studies, we did not exclude hospital admissions for neonatal complications within our cohort (eg, [16]) which could have resulted in an overestimate of some conditions. Additionally, only the primary diagnosis codes were used in the study, apart from cardiac conditions, limiting our findings in relation to comorbid conditions.

It is also important to acknowledge that the prevalence estimates we report relating to specific conditions only apply when a child has been admitted to hospital for such a condition. For some conditions such as leukaemia hospital admission is universal but for other conditions experienced by people with Down syndrome e.g. sleep apnoea, we found a low (5.2%) percentage admitted to hospital. We have previously published on parent-reported prevalence of medical conditions where, in contrast to the hospitalisation data, sleep apnoea was reported in almost 20% of juveniles with Down syndrome [4], [33]. The parent report data are not clinically validated but can provide cross-sectional information on many conditions such as hypothyroidism, constipation and refractive errors, information which would not be available through hospital records. On the other hand the hospital morbidity data provide both a longitudinal perspective and clinically validated information using ICD diagnostic codes on more severe conditions requiring hospitalisation. Both datasets are population-based but provide different perspectives and are complementary.

There are several areas of possible future research stemming from our findings. By assessing the median age of admission, we found, that certain conditions were more likely to occur at selected developmental periods (e.g. cardiac conditions among the very young, middle ear and hearing conditions among early childhood and oral cavity conditions among middle childhood). The need to further investigate changes in hospitalisation patterns among people with Down syndrome as they age therefore remains important. Recently collected data from carers of adolescent children with Down Syndrome will also provide an opportunity to better understand healthcare utilisation in WA among this older group [51] while also capturing health care utilisation for conditions not requiring hospitalisation.

## Conclusion

These findings provide population-level evidence that children with Down syndrome are at increased risk of morbidity early in childhood but to a lesser extent in adolescence and early adulthood. Infections, involving the respiratory, ENT and gastro-intestinal systems were the largest contributor to hospitalisations, affecting nearly 80% of children and accounting for almost one third of admissions. This health burden underscores the increasing importance of comprehensive primary care for this group so that infections can be prevented or identified and treated early before progressing to the need for hospitalisation. Further research is needed to better understand the longer term implications of health conditions upon the young people and their families and how their health patterns change with age.

## References

[1] O'Leary P, Bower C, Murch A, Crowhurst J, Goldblatt J (1996) The impact of antenatal screening for Down syndrome in Western Australia: 1980–1994. Aust and NZ J Obstet Gynaecol 36: 385–388. 10.1111/j.1479-828x.1996.tb02176.x 9006817

[2] O'Leary P, Breheny N, Reid G, Charles T, Emery J (2006) Regional variations in prenatal screening across Australia: Stepping towards a national policy framework. Aust N Z J Obstet Gynaecol 46: 427–432. 16953858 10.1111/j.1479-828X.2006.00629.x

[3] Bower C, Rudy E, Callaghan A, Quick J, Cosgrove P, et al.. (2011) Western Australian Register of Developmental Anomalies1980–2010. PerthWestern Australia, : King Edward Memorial Hospital, Women's and Children's Health Service

[4] Leonard S, Bower C, Petterson B, Leonard H (1999) Medical aspects of school-aged children with Down syndrome. Dev Med Child Neurol 41: 683–688. 10587045 10.1017/s0012162299001401

[5] Cleves MA, Hobbs CA, Cleves PA, Tilford JM, Bird TM, et al (2007) Congenital defects among liveborn infants with Down syndrome. Birth Defects Res A Clin Mol Teratol 79: 657–663. 17696161 10.1002/bdra.20393

[6] Frid C, Anneren G, Rasmussen F, Sundelin C, Drott P (2002) Utilization of medical care among children with Down's syndrome. J Intellect Disabil Res 46: 310–317. 12000582 10.1046/j.1365-2788.2002.00392.x

[7] Irving CA, Chaudhari MP (2012) Cardiovascular abnormalities in Down's syndrome: spectrum, management and survival over 22 years. Arch Dis Child 97: 326–330. 21835834 10.1136/adc.2010.210534

[8] van Trotsenburg AS, Heymans HS, Tijssen JG, de Vijlder JJ, Vulsma T (2006) Comorbidity, hospitalization, and medication use and their influence on mental and motor development of young infants with Down syndrome. Pediatrics 118: 1633–1639. 17015556 10.1542/peds.2006-1136

[9] Määttä T, Kaski M, Taanila A, Iivanainen M (2006) Sensory impairments and health concerns related to the degree of intellectual disability in people with Down syndrome. Downs Syndr Res Prac 11: 78–83. 10.3104/reports.317 17048801

[10] Goldacre MJ, Wotton CJ, Seagroatt V, Yeates D (2004) Cancers and immune related diseases associated with Down's syndrome: a record linkage study. Arch Dis Child 89: 1014–1017. 15499053 10.1136/adc.2003.046219 PMC1719725

[11] Hill DA, Gridley G, Cnattingius S, Mellemkjaer L, Linet M, et al (2003) Mortality and Cancer Incidence Among Individuals With Down Syndrome. Arch Intern Med 163: 705–711. 12639204 10.1001/archinte.163.6.705

[12] Patja K, Pukkala E, Sund R, Iivanainen M, Kaski M (2006) Cancer incidence of persons with Down syndrome in Finland: a population-based study. Int J Cancer 118: 1769–1772. 16231334 10.1002/ijc.21518

[13] Sullivan SG, Hussain R, Glasson EJ, Bittles AH (2007) The profile and incidence of cancer in Down syndrome. J Intellect Disabil Res 51: 228–231. 17300418 10.1111/j.1365-2788.2006.00862.x

[14] Bergholdt R, Eising S, Nerup J, Pociot F (2006) Increased prevalence of Down's syndrome in individuals with type 1 diabetes in Denmark: A nationwide population-based study. Diab tologia 49: 1179–1182. 10.1007/s00125-006-0231-6 16575558

[15] Hilton JM, Fitzgerald DA, Cooper DM (1999) Respiratory morbidity of hospitalized children with Trisomy 21. J Paediatr Child Health 35: 383–386. 10457298 10.1046/j.1440-1754.1999.00386.x

[16] So SA, Urbano RC, Hodapp RM (2007) Hospitalizations of infants and young children with Down syndrome: Evidence from inpatient person-records from a statewide administrative database. J Intellect Disabil Res 51: 1030–1038. 17991010 10.1111/j.1365-2788.2007.01013.x

[17] Thomas K, Bourke J, Girdler S, Bebbington A, Jacoby P, et al (2011) Variation over time in medical conditions and health service utilization of children with Down syndrome. J Pediatr 158: 194–e191, 194-200, e191. 20934710 10.1016/j.jpeds.2010.08.045

[18] Leonard S, Bower C, Petterson B, Leonard H (2000) Survival of infants born with Down's syndrome: 1980–96. Paediatr Perinat Epidemiol 14: 163–171. 10791661 10.1046/j.1365-3016.2000.00252.x

[19] de Hingh YC, van der Vossen PW, Gemen EF, Mulder AB, Hop WC, et al (2005) Intrinsic abnormalities of lymphocyte counts in children with down syndrome. J Pediatr 147: 744–747. 16356423 10.1016/j.jpeds.2005.07.022

[20] Guazzarotti L, Trabattoni D, Castelletti E, Boldrighini B, Piacentini L, et al (2009) T lymphocyte maturation is impaired in healthy young individuals carrying trisomy 21 (Down syndrome). Am J Intellect Dev Disabil 114: 100–109. 19391670 10.1352/2009.114.100-109

[21] Joyce A, Tran BN (2009) Twenty-seventh Annual Report of the Western Australian Midwives' Notification System. Perth.

[22] Petterson B, Leonard H, Bourke J, Sanders R, Chalmers R, et al (2004) IDEA (Intellectual Disability Exploring Answers): A population based database for intellectual disability in Western Australia. Ann Hum Biol 32: 237–243. 10.1080/03014460500075035 16096222

[23] Holman CDAJ, Bass AJ, Rouse IL, Hobbs MST (1999) Population-based linkage of health records in Western Australia: development of a health services research linked database. Aust N Z J Public Health 23: 453–459. 10575763 10.1111/j.1467-842x.1999.tb01297.x

[24] Williams K, Leonard H, Tursan d'Espaignet E, Colvin L, Slack-Smith L, et al (2005) Hospitalisations from birth to 5 years in a population cohort of Western Australian children with intellectual disability. Arch Dis Child 90: 1243–1248. 16301550 10.1136/adc.2004.062422 PMC1720232

[25] Silva D, Palandri G, Bower C, Gill L, Codde J, et al.. (1999) Child and adolescent health in Western Australia: An overview. East Perth, Western Australia: Health Department of Western Australia and TVW Telethon Institute for Child Health Research.

[26] National-Coding-Centre (1996) Australian Version of the International Classification of Diseases, 9th revision, Clinical Modification (ICD-9-CM), Second Edition.

[27] Agresti A (2007) An Introduction to Categorical Data Analysis, Second Edition. HobokenNJ: Wiley.

[28] Klein JM, ML(2003) Survival Analysis: Techniques for Censored and Truncated Data, 2nd Edition. New York: Springer.

[29] StataCorp (2009) Stata Statistical Software: Release 11: College Station, TX: StataCorp LP.

[30] Microsoft Corporation (2010) Excel 2010 (Version 14) Microsoft Corporation.

[31] Silva D, Palandri G, Bower C, Gill L, Codde J, et al.. (1999) Specific child and adolescent health problems in Western Australia. East Perth, Western Australia: Health Department of Western Australia and TVW Telethon Institute for Child Health Research

[32] Schieve LA, Boulet SL, Boyle C, Rasmussen SA, Schendel D (2009) Health of children 3 to 17 years of age with down syndrome in the 1997–2005 national health interview survey. Pediatrics 123: e253–260. 19171577 10.1542/peds.2008-1440

[33] Thomas K, Girdler S, Bourke J, Deshpande A, Bathgate K, et al.. (2010) Overview of Health Issues in School-aged Children with Down Syndrome. In: Urbano R, C., editor. International Review of Research in Mental Retardation: Academic Press. pp. p. 67–106.

[34] Shott SR (2006) Down syndrome: common otolaryngologic manifestations. Am J Med Genet C Semin Med Genet 142C: 131–140. 16838306 10.1002/ajmg.c.30095

[35] Venail F, Gardiner Q, Mondain M (2004) ENT and speech disorders in children with Down's syndrome: an overview of pathophysiology, clinical features, treatments, and current management. Clin Pediatr 43: 783–791. 10.1177/000992280404300902 15583773

[36] Ram G, Chinen J (2011) Infections and immunodeficiency in Down syndrome. Clin Exp Immunol 164: 9–16. 21352207 10.1111/j.1365-2249.2011.04335.x PMC3074212

[37] Driscoll C, Kei J, Bates D, McPherson B (2003) Tympanometry and TEOAE Testing of Children with Down Syndrome in Special Schools. The Australian and New Zealand Journal of Audiology 25: 85–93.

[38] Marcell MM, Ridgeway MM, Sewell DH, Whelan ML (1995) Sentence imitation by adolescents and young adults with Down's syndrome and other intellectual disabilities. J Intellect Disabil Res 39: 215–232. 7640492 10.1111/j.1365-2788.1995.tb00504.x

[39] Roizen NJ, Patterson D (2003) Down's syndrome. The Lancet 361: 1281–1289. 10.1016/S0140-6736(03)12987-X 12699967

[40] Van Cleve S, Cohen W (2006) Part 1: Clinical Practice Guidelines for Children With Down Syndrome From Birth to 12 Years. J Pediatr Health Care 20: 47–54. 16399479 10.1016/j.pedhc.2005.10.004

[41] Slack-Smith L, Colvin L, Leonard H, Kilpatrick N, Bower C, et al (2009) Factors associated with dental admissions for children aged under 5 years in Western Australia. Arch Dis Child 94: 517–523. 19060007 10.1136/adc.2008.145672

[42] Kruger E, Dyson K, Tennant M (2006) Hospitalization of Western Australian children for oral health related conditions: a 5–8 year follow-up. Aust Dent J 51: 231–236. 17037889 10.1111/j.1834-7819.2006.tb00434.x

[43] Milne E, Laurvick CL, de Klerk N, Robertson L, Thompson JR, et al (2008) Trends in childhood acute lymphoblastic leukemia in Western Australia, 1960–2006. Int J Cancer 122: 1130–1134. 17985340 10.1002/ijc.23226

[44] Colvin L, Bower C (2009) A retrospective population-based study of childhood hospital admissions with record linkage to a birth defects registry. BMC Pediatr 9: 32. 19426556 10.1186/1471-2431-9-32 PMC2692976

[45] Geelhoed EA, Bebbington A, Bower C, Deshpande A, Leonard H (2011) Direct Health Care Costs of Children and Adolescents with Down Syndrome. J Pediatr 159: 541–545. 21784457 10.1016/j.jpeds.2011.06.007 PMC3858577

[46] Bloemers BL, van Furth AM, Weijerman ME, Gemke RJ, Broers CJ, et al (2007) Down syndrome: a novel risk factor for respiratory syncytial virus bronchiolitis–a prospective birth-cohort study. Pediatrics 120: e1076–1081. 17908728 10.1542/peds.2007-0788

[47] Zeller B, Gustafsson G, Forestier E, Abrahamsson J, Clausen N, et al (2005) Acute leukaemia in children with Down syndrome: a population-based Nordic study. Br J Haematol 128: 797–804. 15755283 10.1111/j.1365-2141.2005.05398.x

[48] Petterson B, Leonard H, Bourke J, Sanders R, Chalmers R, et al (2005) IDEA (Intellectual Disability Exploring Answers): A population-based database for intellectual disability in Western Australia. Ann Hum Biol 32: 237–243. 16096222 10.1080/03014460500075035

[49] Kupferman JC, Druschel CM, Kupchik GS (2009) Increased prevalence of renal and urinary tract anomalies in children with Down syndrome. Pediatrics 124: e615–621. 19752083 10.1542/peds.2009-0181

[50] Zachariah P, Ruttenber M, Simões EA (2012) Down Syndrome and Hospitalizations due to Respiratory Syncytial Virus: A Population-Based Study. J Pediatr 160: 827–831. 22177993 10.1016/j.jpeds.2011.11.004

[51] Foley KR, Jacoby P, Girdler S, Bourke J, Pikora T, et al (In press) Functioning and post-school transition outcomes for young people with Down syndrome. Child Care Health Dev 10.1111/cch.12019 23294187

